# *Acacia* Changes Microbial Indicators and Increases C and N in Soil Organic Fractions in Intercropped *Eucalyptus* Plantations

**DOI:** 10.3389/fmicb.2018.00655

**Published:** 2018-04-04

**Authors:** Arthur P. A. Pereira, Maurício R. G. Zagatto, Carolina B. Brandani, Denise de Lourdes Mescolotti, Simone R. Cotta, José L. M. Gonçalves, Elke J. B. N. Cardoso

**Affiliations:** ^1^Soil Microbiology Laboratory, Department of Soil Science, Luiz de Queiroz College of Agriculture, University of São Paulo, Piracicaba, Brazil; ^2^Department of Forest Sciences, Luiz de Queiroz College of Agriculture, University of São Paulo, Piracicaba, Brazil

**Keywords:** forest soil, soil biology, mixed-systems, C-N cycles, organic matter

## Abstract

Intercropping forest plantations of *Eucalyptus* with nitrogen-fixing trees can increase soil N inputs and stimulate soil organic matter (OM) cycling. However, microbial indicators and their correlation in specific fractions of soil OM are unclear in the tropical sandy soils. Here, we examined the microbial indicators associated with C and N in the soil resulting from pure and intercropped *Eucalyptus grandis* and *Acacia mangium* plantations. We hypothesized that introduction of *A. mangium* in a *Eucalyptus* plantation promotes changes in microbial indicators and increases C and N concentrations on labile fractions of the soil OM, when compared to pure eucalyptus plantations. We determined the microbial and enzymatic activity, and the potential for C degradation by the soil microbial community. Additionally, we evaluated soil OM fractions and litter parameters. Soil (0–20 cm) and litter samples were collected at 27 and 39 months after planting from the following treatments: pure *E. grandis* (E) and *A. mangium* (A) plantations, pure *E. grandis* plantations with N fertilizer (E+N) and an *E. grandis*, and *A. mangium* intercropped plantations (E+A). The results showed that intercropped plantations (E+A) increase 3, 45, and 70% microbial biomass C as compared to A, E+N, and E, at 27 months after planting. The metabolic quotient (*q*CO_2_) showed a tendency toward stressful values in pure *E. grandis* plantations and a strong correlation with dehydrogenase activity. A and E+A treatments also exhibited the highest organic fractions (OF) and C and N contents. A canonical redundancy analysis revealed positive correlations between microbial indicators of soil and litter attributes, and a strong effect of C and N variables in differentiating A and E+A from E and E+N treatments. The results suggested that a significant role of *A. mangium* enhance the dynamics of soil microbial indicators which help in the accumulation of C and N in soil OF in intercropped *E. grandis* plantations. Our results are mostly relevant to plantations in sandy soil areas with low levels of OM, suggesting and efficient method for improving nutrient availability in the soil and optimizing eucalyptus growth and development.

## Introduction

Brazil is the world's largest producer of *Eucalyptus* spp., a species of fundamental ecological, social, and financial importance due to its effects in reducing pressures on native forests and generating direct and indirect jobs (ABRAF, 2013). However, the sustainability of *Eucalyptus* plantations has been intensely debated due to their concentration in south and central Brazil, where low fertility soils prevail, and *Eucalyptus* is mostly grown in mono-specific plantations (Gonçalves et al., 2013). Additionally, *Eucalyptus* production areas in Brazil have relatively short-rotation (6–7 years) and high productivity (~37 m^−3^ ha^−1^ year^−1^), which entails a high nutrient exportation in the harvest and a negative balance in soil nitrogen over time (Bouillet et al., 2008; Gonçalves et al., 2013; Pulito et al., 2015).

As a result, mineral fertilizers are employed to maintain productivity levels of *Eucalyptus* plantations, increasing both economic input and risk of environmental pollution. In this context, intercropped *Eucalyptus grandis* and *Acacia mangium* plantations represent a convenient forest management strategy as it minimizes environmental side-effects especially in sandy soils with low organic matter (OM) levels (Laclau et al., 2008). *A. mangium* (family Fabaceae) forms associations with the rhizobia diazotrophic bacteria, promoting greater N availability in the soil. Several studies have revealed improvements generated by this association, especially in C and N dynamics, and wood productivity (Bouillet et al., 2008; Laclau et al., 2008; Voigtlaender et al., 2012; Paula et al., 2015; Fonseca et al., 2017).

N inputs promoted by *Acacia* can improve biogeochemical cycles and enhance the ecological intensification of ecosystem services (Bouillet et al., 2008; Laclau et al., 2008; Richards et al., 2010; Forrester et al., 2011; Bini et al., 2013; Rachid et al., 2013). Therefore, a better understanding of changes in soil microbial indicators and C and N dynamics in sandy soils is especially important in an intercropped plantation design, as they may reflect OM quality and soil nutrients availability and reduce fertilizer use (Bouillet et al., 2008; Voigtlaender et al., 2012; Cardoso et al., 2013; Pereira et al., 2017). However, little is known about soil microbial indicators in either pure or intercropped *E. grandis* and *A. mangium* plantations in sandy soils.

Bini et al. (2013) published the first evaluation of microbial attributes in an intercropped system at 2, 7, 14, and 20 months after planting, but results were highly variable across times. Additionally, the authors did not highlight the effects of *A. mangium* on enzyme activity associated with C and N cycling in soil, the potential for degradation of C sources, and the reflexes of these indicators on C and N concentrations in soil OM fractions. Thus, important questions remain unanswered such as the potential effect of introduction of *A. mangium* in an intercropped system on soil microbial attributes and soil OM labile fractions. Investigating those questions is crucial to planning strategies of *Eucalyptus* plantations and their sustainability in tropical soils.

Here, we evaluate changes in soil microbial quality indicators such as microbial activity and enzymatic and metabolic potential associated with C and N cycles in the soil. Additionally, we characterize soil OM fractions and litter attributes and examine their correlations, in order to further explore the microbial influence on C and N cycling and how it can discriminate among plantation treatments. We hypothesized that intercropping of *A. mangium* in *E. grandis* plantations can promote changes in microbial indicators (microbial, enzymatic, and metabolic activity) and increase C and N concentrations on labile fractions of soil OM.

## Materials and methods

### Experimental design and sampling

#### Location, climate, and soil classification

**Figure 1:**
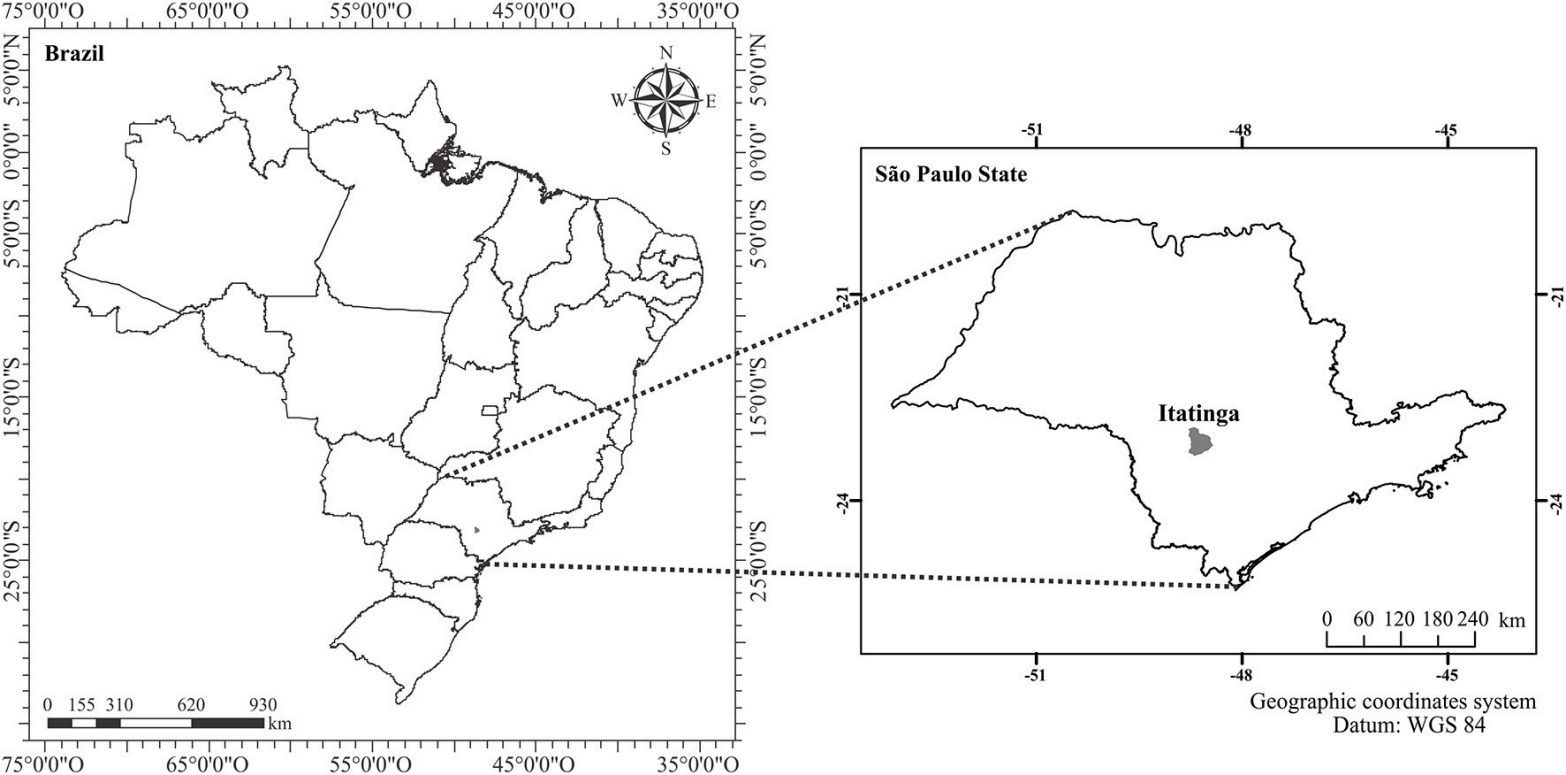
Geographic location of Itatinga municipality, São Paulo state, Brazil.

The study was carried out at the Itatinga Forest Sciences Experimental Station (23°03′00″S−48°37′00″W; 830 m above sea level), Department of Forest Sciences, Luiz de Queiroz College of Agriculture, University of São Paulo, Itatinga, São Paulo state, Brazil (Figure 1). The climate in this region is Cfa (Köppen classification), with an annual rainfall of 1,350 mm mostly concentrated (75%) between March and October (Laclau et al., 2008). The soil was classified as red-yellow Latosol (Brazilian soil classification system) or Ferralsol (FAO/WRB) of sandy texture, low cation exchange capacity (31.6 cmol_c_ dm^−3^) and typically dystrophic, with the following mean available nutrient levels in the 0–20 cm layer: P = 4.6 mg dm^−3^, K^+^ = 0.7, Ca^2+^ = 8.6, Mg^2+^ = 7.91, and Al^3+^ = 14 cmol_c_ dm^−3^ (van Raij et al., 2001; Empresa Brasileira de Pesquisa Agropecáuária, 2013).

#### Soil history, experimental design, planting, and tree fertilization

The experiment was based on a first rotation installed in December 2013 in an area previously planted with *E. grandis* (30–50 years) and managed without fertilizer application. After clearing the vegetation, the experiment was implemented as a fully randomized block design with the following four treatments: pure *E. grandis* (E) and *A. mangium* (A) plantations, pure *E. grandis* plantations with N fertilizer (E+N), and *E. grandis* and *A. mangium* intercropped plantations (E+A), with four blocks each and totaling 16 plots. Trees were planted in a minimum tillage system with 3 × 3 m spacing (9 m^2^ per plant). Total area of plots was 1,296 m^2^ (36 × 36 m), and useful area eliminating the edge effect was 576 m^2^ (24 × 24 m). The intercropped plantation (E+A) was in double rows, with a plant ratio of 1:1. *A. mangium* seedlings were inoculated with *Rhizobium* strains previously selected by Embrapa Agrobiology for their high biological N-fixing capacity and high nodulation rates in *Acacia* spp. Nitrogen fertilization in the treatment E+N was carried out in December 2013 and 2014, with the application of 10 and 90 kg ha^−1^ of N respectively, using 450 kg ha^−1^ as ammonium sulfate (NH_4_)_2_SO_4_. Other nutrients applied to all treatments can be found in Table S1.

#### Soil and litter sampling

In March 2016 and 2017, corresponding respectively to the 27th and 39th months of the trees, we sampled 120 soil samples (0–20 cm) by drawing a Voronoi polygon, the elementary space defined by the half distances between the sampled tree and its neighbors for each tree (Figure S1; Honda, 1978; Saint-André et al., 2005). We collected samples from six representative trees in each experimental plot, totalizing six subsamples per plot, which were homogenized and mixed into a composite sample (Figure S1). Twelve subsamples were taken from mixed treatments E+A (six trees of *A. mangium* and six of *E. grandis*), which were homogenized into a composite sample. The sampled litter (included twigs, branches, and leaves) followed the same standardization procedure. However, we used a template (25 × 25 cm) placed on the soil surface and sampled all organic material underneath. As forest rotations in Brazil take about 6 or 7 years, we selected our sampling periods in order to assess differences between the beginning (27 months) and the maximum (39 months) period of litter deposition in the soil, and to contrast possible differences in C and N cycling in the soil.

For microbiological analyses, soil was sieved (2 mm) and stored at 4°C until the analyses. For chemical and physical analyses soil was sieved (2 mm) and air dried for 72 h. Litter was oven-dried at 60°C for 24 h and then ground (1 mm) for chemical analyses.

### Analytical procedures

#### Microbial indicators analyses

##### Microbial biomass, respiration, and enzymatic activity

Microbial biomass of C (Cmic) and N (Nmic) were estimated by the fumigation-extraction method, using the coefficients K_c_ = 0.40 and K_n_ = 0.54, respectively (Brookes et al., 1985; Vance et al., 1987). Soil respiration (SR) was estimated by quantification of CO_2_-C emitted during 28 days of incubation at 28°C (Bonfim et al., 2016). In both tests, soil moisture was maintained at 60% of maximum water retention capacity. The relationship between SR and Cmic was used to calculate the metabolic quotient (*qCO*_2_) (Anderson and Domsch, 1993). In addition, C and N microbial quotients (*qMic-C* and *qMic-N*, respectively) were calculated using the relationship between Cmic and total organic carbon (TOC) (Cmic/TOC) and Nmic and total soil N (Nmic/Total-N) (Bini et al., 2014).

Potential of urease (EC 3.5.1.5), L-asparaginase (EC 3.5.1.1), L-glutaminase (EC 3.5.1.2), amidase (EC 3.5.1.4), β-D-glucosidase (EC 3.2.1.21), and dehydrogenase (EC 1.1.1.) activity were determined following Tabatabai (1994). Due to the short time of incubation (1–2 h), toluene was omitted from the analysis (Souza et al., 2016). The six soil enzymes were selected for their participation in C (β-D-glucosidase and dehydrogenase) and N (urease, L-asparaginase, L-glutaminase and amidase) cycling, respectively. All assays were performed in duplicates.

##### Metabolic profile of the soil microbial community

The potential of soil microbial communities in the degradation of C sources was assessed with Biolog® EcoPlate (Zak et al., 1994). This approach uses colorimetric detection (tetrazolium dye as redox indicator) to measure microbial activity in 31 different C sources, including carbohydrates, polymers, carboxylic acids, amines, amino acids, and miscellaneous compounds. Microbial suspensions were prepared using 5 g of fresh soil from each sample in 45 ml of sterile saline solution (0.85% NaCl) and shaking at 150 rpm during 30 min. Soil suspension was subjected to tenfold dilutions in sterilized saline solution to a final dilution of 10^−2^. An aliquot (150 μL) was added to each plate well, using one-third of the plate for each compost sample replicate. The plates were incubated at 25°C and inspected every 24 h for 6 days at 590 nm by an ELISA plate reader. The potential of soil microbial community in the degradation of C sources was used to calculate the community niche (CN) index in each treatment. We calculated the CN index by summing over the 31 C sources and the maximal potential observed for the microbial community in each soil sample and individual C source (Salles et al., 2009).

#### Soil and litter analyses

To evaluate the influence of microbial indicators on the dynamics of C and N, soil OM was physically fractionated from a soil air-dried mass (20 g) using the granulometric method (Brandani et al., 2017). This process, separates organic (OF) (2,000–75 μm), organic-mineral 1 (OMinF1) (2,000–75 μm), organic-mineral 2 (OMinF2) (75–53 μm) and organic-mineral 3 (OMinF3) (<53 μm) fractions. C and N concentrations in each OM fraction were determined by dry combustion with an elemental analyzer (LECO®) (Nelson and Sommers, 1982; Christensen, 2001; Signor et al., 2014). TOC and Total-N contents in soil OM fractions were obtained by multiplying C and N concentrations of each fraction by their corresponding mass (Brandani et al., 2017).

Litter was dried at 60°C for 24 h and ground (1 mm). Total C and N were determined equally by dry combustion in the elemental analyzer (LECO®). In addition, ${\text{NH}}_{4}^{+}$-N and ${\text{NO}}_{3}^{-}$N fractions were extracted by nitric-perchloric digestion and contents were determined using the “Kjeldahl” method (Nelson and Sommers, 1982; Vezzani et al., 2001; Freitas et al., 2013).

### Statistical analyses

Data were examined for homogeneity-normality of variances by Levene and Shapiro–Wilk's tests. The data set was analyzed through one-way ANOVA and groups means were compared through Tukey's tests at a significance level of 5%. A Canonical Redundancy Analysis (RDA) was performed to identify variables discriminating among treatments (Baretta et al., 2008). In RDA, microbiological indicators (microbial and enzymatic activity) were correlated with soil and litter variables to identify patterns across treatments. In parallel, Monte Carlo test was performed by considering 499 random permutations and displaying “*p*” values and Wilk's lambda (%) of the attributes, resulting in estimates of significance and weight of each correlation (Baretta et al., 2008). Due to collinearity, factors with an inflation factor >20 were removed (Baretta et al., 2008). A heat map and a Principal Coordinates Analysis (PCoA) were performed to characterize the metabolic profile of the soil microbial community, revealing the distribution of consumption across the 31 C sources. An ANOSIM test was applied to identify differences between the treatments showed in PCoA (Ramette, 2007). Univariate analyses and the heat map graphic were prepared using R software (https://www.r-project.org, using “agricolae,” “gplots,” and “RColorBrewer” packages), and multivariate analysis was performed on Canoco® software for Windows (v.4.5) and PRIMER-E (Leps and Smilauer, 2003; Baretta et al., 2008).

## Results

### Microbial biomass C and N contents, and soil microbial activity

**Figure 2:**
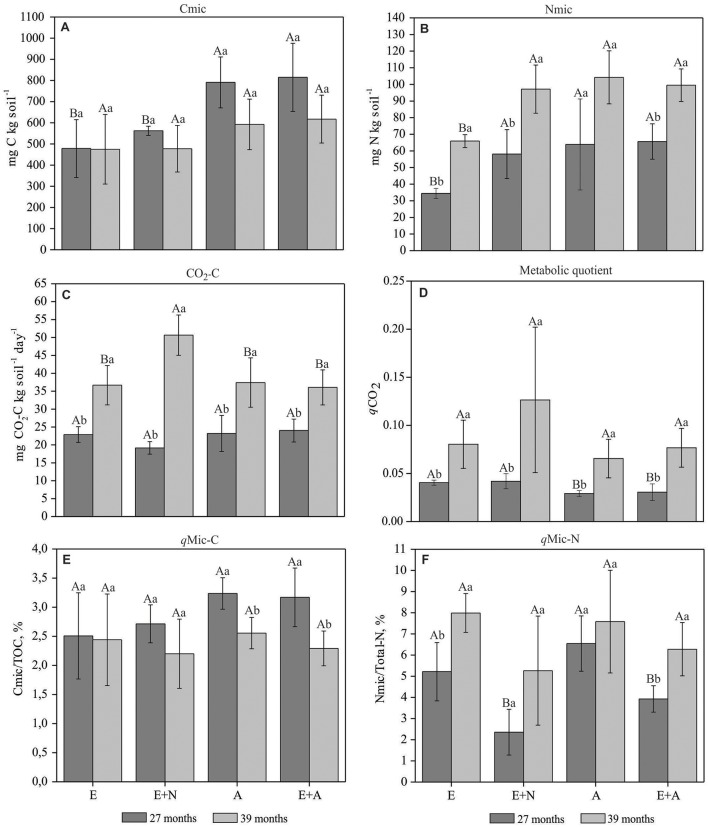
Biological soil indicators in pure and mixed *E. grandis* and *A. mangium* plantations. (E) *E. grandis*, (E+N) *E. grandis* with N fertilization, (A) *A. mangium*, and (E+A) mixed plantation of *E. grandis* and *A. mangium* at 27 and 39 months after planting. **(A)** Cmic: microbial C; **(B)** Nmic: microbial N; **(C)** CO_2_-C: Soil Respiration; **(D)** *q*CO_2_: Metabolic quotient; **(E)** *q*Mic-C: Microbial C quotient, and **(F)** *q*Mic-N: Microbial N quotient. Means followed by the same letter do not differ by Tukey's test at a significance level of 5%. Upper case letters compared treatments within each period and lower case letters compared periods within each treatment. Error bars indicate standard deviation; *n* = 4.

Microbial C content (Cmic) was significantly higher in A and E+A stands and lower in E and E+N stands at 27 months after planting, when mixed stands E+A showed increases of 45 and 70% in soil Cmic compared E+N and E, respectively, and almost similar to A (increase of only 3%) (*p* < 0.05) (Figure 2A). In contrast, at 27 months microbial N content (Nmic) did not differ between E+N, A and E+A stands, but E stands shows the lowest soil Nmic content. E stands also showed the lowest Nmic content at 39 months. In general, soil Nmic contents were highest at 39 months, except in A stands that did not show significant differences at any period. Increases in soil Nmic contents between 27 and 39 months after planting in E, E+N, and E+A stands were, respectively 68, 92, and 51% (*p* < 0.05; Figure 2B).

Soil CO_2_-C emission rates did not vary between treatments at 27 months, with a mean value of 22 mg CO_2_-C kg^−1^ day^−1^. At 39 months CO_2_-C emissions had significantly increased in all treatments, especially in the E+N treatment where rates more than doubled (50.5 mg CO_2_-C kg^−1^ day^−1^) (*p* < 0.05; Figure 2C). Metabolic quotient (*q*CO_2_) values increased with time in all treatments (*p* < 0.05) (Figure 2D). There was a non-significant trend toward higher values between 27 and 39 months in E and E+N stands.

The C microbial index (*q*Mic-C) was significantly higher at 27 months for A and E+A treatments, but not for E stands. The N microbial index (*q*Mic-N) at 27 months was higher in the E and A treatments (*p* < 0.05; Figure 2F). N fertilization in *Eucalyptus* (E+N) decreased *q*Mic-N to half the value of E stands at 27 months after planting. At 39 months *q*Mic-N was significantly higher for all treatments except A (*p* < 0.05; Figure 2F).

### Potential activity of soil enzymes

**Figure 3:**
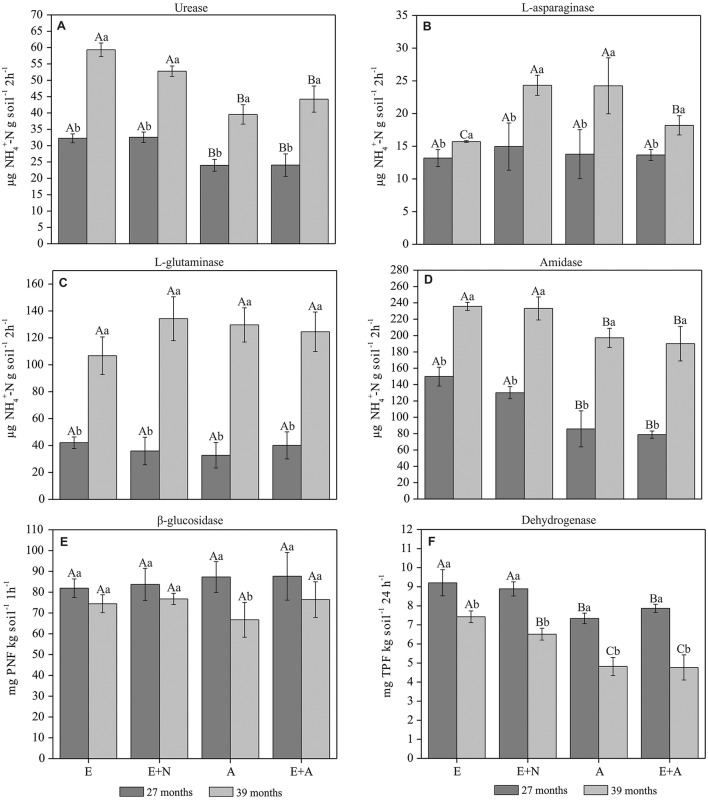
Potential enzymatic soil activity in pure and mixed *E. grandis* and *A. mangium* plantations. (E) *E. grandis*, (E+N) *E. grandis* with N fertilization, (A) *A. mangium*, and (E+A) mixed plantation of *E. grandis* and *A. mangium* at 27 and 39 months after planting. **(A)** Urease; **(B)** L-asparaginase; **(C)** L-glutaminase; **(D)** Amidase; **(E)** β-glucosidase, and **(F)** Dehydrogenase. Means followed by the same letter do not differ by Tukey's test at a significance level of 5%. Upper case letters compared treatments within each period and lower-case letter compared periods within each treatment. Error bars indicate standard deviation; *n* = 4.

Potential activity of urease was significantly higher in E and E+N treatments in both periods. However, the potential of urease activity was 84, 62, 65, and 84% higher at 39 months in E, E+N, A, and E+A, respectively, compared to 27 months (*p* < 0.05; Figure 3A). There were no significant differences in L-asparaginase activity across treatments at 27 months, with a mean value of 13.8 μg ${\text{NH}}_{4}^{+}$-N g^−1^ 2h^−1^. However, the L-asparaginase potential activity was generally higher at 39 months, especially in the E+N and A treatments. Thus, the L-asparaginase potential activity increased by 19, 63, 76, and 33%, respectively in E, E+N, A, and E+A treatments at 39 months (*p* < 0.05; Figure 3B). L-glutaminase activity did not differ among treatments at 27 months after planting. However, there was higher L-glutaminase activity at 39 months, with an increase of ~228% in comparison to 27 months (*p* < 0.05; Figure 3C). The pattern of amidase potential activity was similar to that of urease, with higher activity in E and E+N treatments and lower in A and E+A in both periods. Nevertheless, enzyme activity was 57, 79, 130, and 14% higher in the E, E+N, A, and E+A treatments, respectively at 39 months compared to 27 months (*p* < 0.05; Figure 3D). β-glucosidase activity did not differ among treatments in either period, with mean values of 85.1 and 73.58 mg PNF kg^−1^ h^−1^, respectively. However, when comparing the same treatment between periods, A showed decreased activity in β-glucosidase activity (*p* < 0.05; Figure 3E). Dehydrogenase activity was higher in E and E+N stands, especially at 27 months. At 39 months, there were also differences in the dehydrogenase activity potential (E > E+N > A = E+A; Figure 3F).

### Microbial metabolic profile

**Figure 4:**
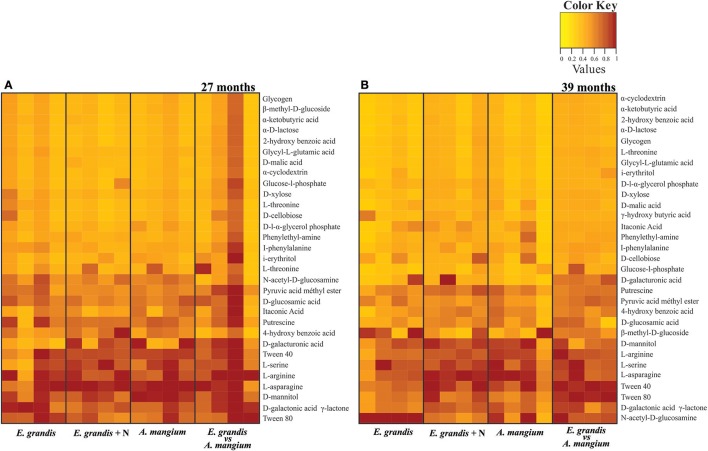
Metabolic profile showed the potential of 31 C sources degradation assessed by the Biolog EcoPlates in pure and mixed *E. grandis* and *A. mangium* plantations at **(A)** 27 and **(B)** 39 months after planting the trees. The score in a heat map analysis represents the difference between the consumption of each sample for the same substrate. The highest consumption can be identified by a red color and the lowest consumption by a yellow color.

At 27 and 39 months after tree planting, there were no significant differences between treatments regarding the potential of the 31 C sources degradation (Table S2 and Figures 4A,B). However, we verified significant differences when comparing periods, mainly in A (16 sources) and E+A (5 sources; Table S2 and Figure 4). The PCoA analysis and the ANOSIM test did not show significant differences between the treatments within the same period (Figures S2A,B). However, was detected significant separation between 27 and 39 months after planting, only for the E+A treatment (*R* = 0.795, *p* < 0.0247) Figure S2C. The community niche index (CN) did not show significant differences between treatments, although it showed a slight increase in A and E+A at 27 months. However, there was a significant effect between the sampling times, where treatments A and E+A showed a reduction in the CN index at the 39 months (*p* < 0.05; Figure S4).

### Organic matter fractions and litter attributes

Organic fraction (OF) presented double the mass in *A. mangium* (A) and mixed stands (E+A) when compared to other treatments, especially at 39 months (*p* < 0.05; Table 1). Most of the initial soil mass analyzed (20 g) was found in the OMinF2 fraction (organo-mineral fraction 75–53 μm), which ranged from 63 to 70% and did not present significant statistical differences across treatments (*p* < 0.05; Table 1).

**Table 1:** Physical characterization and C-N concentrations of soil organic matter (0–20 cm) in (E) *E. grandis*, (E+N) *E. grandis* with N addition, (A) *A. mangium*, and (E+A) mixed plantation between *E. grandis* and *A. mangium* at 27 and 39 months after planting.

	**E**		**E**+**N**		**A**		**E**+**A**	
	**27 months**	**39 months**	**27 months**	**39 months**	**27 months**	**39 months**	**27 months**	**39 months**
**OM FRACTIONS MASS (g kg**^−1^**)**								
OF	12.7 ± 1.8^Ba^	14.93 ± 3.4^Ba^	12.91 ± 1.2^Bb^	18.47 ± 0.6^Ba^	25.44 ± 2.1^Ab^	29.55 ± 0.35^Aa^	25.6 ± 2.1^Ab^	30 ± 0.84^Aa^
OMinF1	12.48 ± 20^ns^	12.42 ± 2.69^ns^	13.89 ± 13.6^ns^	10.0 ± 2.19^ns^	13.31 ± 1.78^ns^	10.09 ± 0.46^ns^	12.5 ± 2.4^ns^	10.4 ± 1.30^ns^
OMinF2	700 ± 55.8^ns^	678.2 ± 59.6^ns^	601.1 ± 53.3^ns^	660.2 ± 66.1^ns^	662.8 ± 20.6^ns^	697.59 ± 52.4^ns^	679.1 ± 92.2^ns^	668.7 ± 66.3^ns^
OMinF3	268.1 ± 46.8^ns^	278.94 ± 55.3^ns^	327.3 ± 61.2^ns^	285 ± 44^ns^	288.2 ± 43.6^ns^	223.6 ± 51.8^ns^	286 ± 56.3^ns^	266.6 ± 68.76^ns^
Mean recovery (%)	97	98	97	97	97	96	97	98
**N-CONCENTRATIONS (g kg**^−1^**)**								
N-OF	0.16 ± 0.02^Ba^	0.20 ± 0.05^Ba^	0.17 ± 0.01^Ba^	0.22 ± 0.04^Ba^	0.35 ± 0.04^Aa^	0.35 ± 0.05^Aa^	0.34 ± 0.05^Aa^	0.36 ± 0.06^Aa^
N-OMinF1	0.01 ± 0.004^ns^	0.01 ± 0.008^ns^	0.01 ± 0.003^ns^	0.01 ± 0.01^ns^	0.02 ± 0.01^ns^	0.01 ± 0.003^ns^	0.02 ± 0.01^ns^	0.02 ± 0.006^ns^
N-OMinF2	1.51 ± 0.37^Aa^	0.92 ± 0.69^Aa^	1.25 ± 0.16^Aa^	0.91 ± 0.79^Aa^	1.41 ± 0.21^Aa^	0.80 ± 0.64^Aa^	1.38 ± 0.30^Aa^	1.40 ± 0.23^Aa^
N-OMinF3	0.15 ± 0.02^ns^	0.16 ± 0.05^ns^	0.16 ± 0.02^ns^	0.15 ± 0.03^ns^	0.16 ± 0.02^ns^	0.09 ± 0.01^ns^	0.14 ± 0.07^ns^	0.14 ± 0.04^ns^
Total-N	1.85^Aa^	1.31^Bb^	1.60^Ba^	1.31^Bb^	1.95^Aa^	1.27^Bb^	1.89^Aa^	1.93^Aa^
**C-CONCENTRATIONS (g kg**^−1^**)**								
C-OF	4.82 ± 0.75^Ba^	6.0 ± 1.54^Ba^	5.27 ± 0.45^Ba^	6.60 ± 0.96^Ba^	10.2 ± 1.29^Aa^	9.80 ± 1.49^Aa^	9.97 ± 1.16^Aa^	10.44 ± 1.98^Aa^
C-OMinF1	0.71 ± 0.11^ns^	0.84 ± 0.32^ns^	0.79 ± 0.19^ns^	0.78 ± 0.35^ns^	0.90 ± 0.34^ns^	0.57 ± 0.07^ns^	0.84 ± 0.30^ns^	0.75 ± 0.16^ns^
C-OMinF2	35.3 ± 10.72^ns^	20.05 ± 17.10^ns^	28.91 ± 3.16^ns^	20.18 ± 17.9^ns^	32.77 ± 2.30^ns^	18.02 ± 15.01^ns^	32.24 ± 10.7^ns^	32.27 ± 5.87^ns^
C-OMinF3	0.87 ± 0.65^ns^	0.64 ± 0.12^ns^	0.76 ± 0.39^ns^	0.85 ± 0.16^ns^	0.83 ± 0.23^ns^	0.66 ± 0.32^ns^	0.70 ± 0.26^ns^	0.67 ± 0.14^ns^
TOC	41.71^Aa^	27.54^Bb^	35.75^Ba^	28.43^Bb^	44.7^Aa^	29.07^Bb^	43.77^Aa^	44.15^Aa^

*Means followed by the same letter do not differ by Tukey test at a significance level of 5%. Capital letters, in the line, compared treatments within each period and lower-case letter, in the line, compared periods within each treatment. Mean Standard Deviation (±); n = 4. ns, not significant; OM, Organic Matter; N, Nitrogen; C, Carbon; OF, Organic Fraction (2,000–75 μm); OMinF1, Organic Mineral Fraction 1 (2,000–75 μm); OMinF2, Organic Mineral Fraction 2 (75–53 μm); OMinF3, Organic Mineral Fraction 3 (< 53 μm); TOC, Total Organic Carbon*.

The A and E+A treatments exhibited higher N contents in the OF (2,000–75 μm) than in E and E+N stands, even though the N fertilizer dose in the E+N treatment was 100 kg ha^−1^ of N. Mixed plantations (E+A), when compared to pure plantations E and E+N, showed increases of 45 and 50% (at 27 months) and 55 and 61% (at 39 months) in N contents in the OF respectively (*p* < 0.05). Total-N concentration (Total-N) did not differ at 27 months except for the E+N treatment, which presented the lowest value. On the other hand, at 39 months the E+A treatment showed the highest content of Total-N. Total-N concentrations were always higher at 27 months except for E+A treatment, pointing to a sampling period effect.

Higher C contents were found in A and E+A stands in OF. The mixed stand E+A showed increases of 48 and 53% (at 27 months), and 57 and 63% (at 39 months) of C in OF, when compared to the E and E+N treatments respectively (*p* < 0.05). Total soil C (TOC) did not differ at 27 months except for the E+N treatment that showed the lowest value. The highest concentrations of TOC were detected at 27 months, except for the E+A treatment that did not show differences between sampling periods, although it presented higher soil TOC at 39 months.

Total-N contents in litter were significantly higher in the A stand, especially at 39 months after tree planting. E+A stands displayed lower Total-N content than A stands. However, A stands showed higher contents (*p* < 0.05) than E and E+N stand (especially at 39 months). A and E+A stands exhibited the lowest litter C/N ratios, with the smallest values in the A stand at 39 months (*p* < 0.05) (Table 2). Litter ${\text{NH}}_{4}^{+}$-N was higher in A and E+A, followed by E and E+N stands, which in general exhibited highest values at 27 months.

**Table 2:** Litter chemical characterization in (E) *E. grandis*, (E+N) *E. grandis* with N fertilization, (A) *A. mangium*, and (E+A) mixed plantation between *E. grandis* and *A. mangium* at 27 and 39 months after planting.

	**E**		**E**+**N**		**A**		**E**+**A**	
	**27 months**	**39 months**	**27 months**	**39 months**	**27 months**	**39 months**	**27 months**	**39 months**
Total-C (g kg^−1^)	521 ± 15^Aa^	518 ± 7^Aa^	526 ± 6.5^Aa^	519 ± 20^Aa^	514 ± 9^Aa^	517 ± 8^Aa^	527 ± 9^Aa^	519 ± 3.5^Aa^
Total-N (g kg^−1^)	8.4 ± 0.5^Ca^	9.1 ± 0.3^Ca^	8.5 ± 0.7^Cb^	10.3 ± 0.4^Ca^	14 ± 0.4^Ab^	16.6 ± 1.2^Aa^	11.5 ± 0.1^Bb^	13.7 ± 1.2^Ba^
C/N ratio	62 ± 4.5^Aa^	58 ± 4.83^Aa^	62 ± 5.5^Aa^	51 ± 1.2^Bb^	37 ± 1.8^Ca^	31 ± 2.0^Cb^	46 ± 1, 1^Ba^	39 ± 3.4^Bb^
${\text{NH}}_{4}^{+}$-N (mg kg^−1^)	95.2 ± 4.3^Ba^	53.2 ± 14.5^Ab^	60 ± 4.1^Ca^	36.2 ± 2.4^Ab^	145.6 ± 6^Aa^	22.1 ± 5^Bb^	149 ± 3.3^Aa^	22.4 ± 6.7^Bb^
${\text{NO}}_{3}^{-}$-N (mg kg^−1^)	40 ± 7^ns^	95.2 ± 26^ns^	42 ± 3^ns^	86.1 ± 27^ns^	56.3 ± 3.2^ns^	36.8 ± 5.9^ns^	69 ± 3.9^ns^	17.9 ± 6.7^ns^

*Means followed by the same letter do not differ by Tukey test at a significance level of 5%. Capital letters, in the line, compared treatments within each period and lower-case letter, in the line, compare periods within each treatment. Mean Standard Deviation (±); n = 4. Total-C, Total Carbon; Total-N, Total Nitrogen; C/N, relationship between C and N concentrations; ${\text{NH}}_{4}^{+}$-N, ammonium ion; ${\text{NO}}_{3}^{-}$N, nitrate ion*.

### Global results by redundancy analysis (RDA)

**Figure 5:**
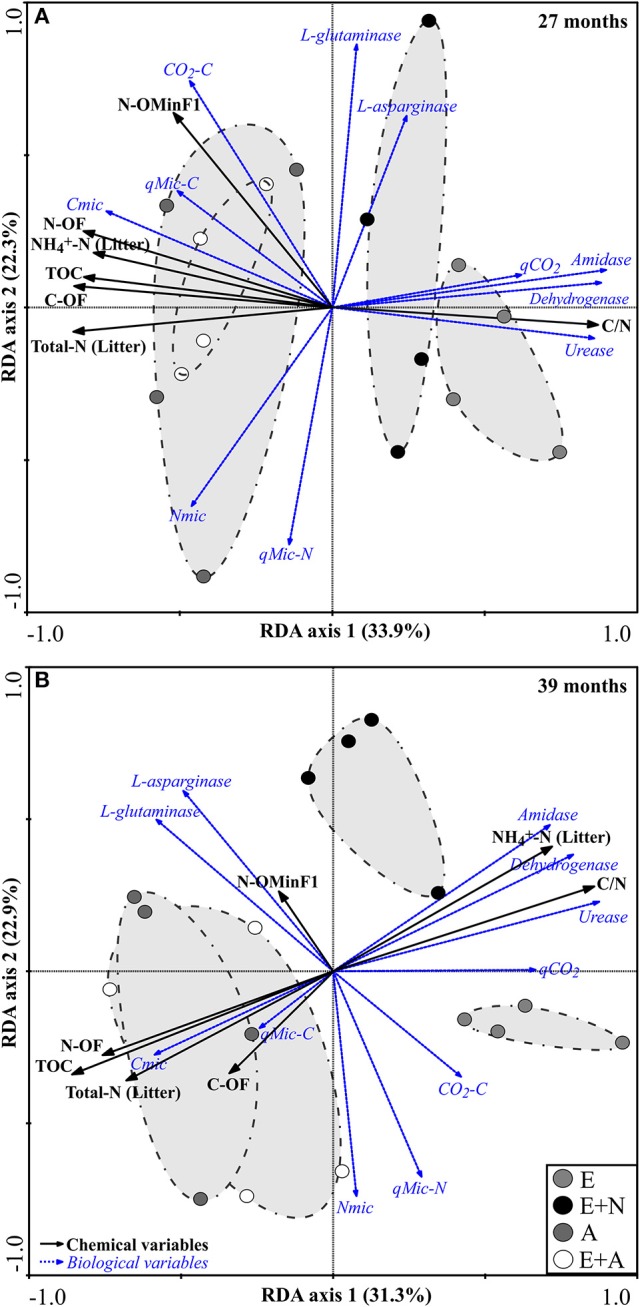
Redundancy analysis (RDA) of soil-litter attributes and soil microbial attributes based on Monte Carlo permutation test (499 permutations). (E) *E. grandis*, (E+N) *E. grandis* with N addition, (A) *A. mangium*, and (E+A) mixed plantation of *E. grandis* and *A. mangium*. Black arrows indicate soil and litter attributes, and blue arrows indicate microbiological attributes. Only significant correlations were fitted in the ordination (*p* < 0.0067). **(A)** 27 months, **(B)** 39 months; *n* = *4*.

At 27 months, RDA revealed a strong differentiation between *Acacia* (A and E+A) and *Eucalyptus* treatments (E and E+N). The first two canonical axes are responsible for 56.2% of variance (33.9% by axis 1 and 22.3% by axis 2). The vectors that discriminated *A. mangium* (A) and mixed system (E+A) from other treatments were the microbial indicators Cmic (λ = 5%, *p* < 0.0039), CO_2_-C (λ = 2%, *p* < 0.0039) and *q*Mic-C (λ = 1%, *p* < 0.0410), which had a strong effect (positive correlation) on soil and litter attributes including C-OF (λ = 14%, *p* < 0.0017), N-OF (λ = 10%, *p* < 0.0018), N-OMinF1 (λ = 9%, *p* < 0.0010), TOC (λ = 2%, *p* < 0.0280), Total-N (Litter) (λ = 17%, *p* < 0.0019), and ${\text{NH}}_{4}^{+}$-N (Litter) (λ = 10%, *p* < 0.0011). On the other hand, the attributes that most contributed to differentiation of *E. grandis* treatments (E and E+N) were the microbial indicators *q*CO_2_ (λ = 2%, *p* < 0.0441), amidase (λ = 9%, *p* < 0.0019) and urease (λ = 1%, *p* < 0.0501), which were positively correlated with litter C/N ratio (λ = 3%, *p* < 0.001) and dehydrogenase (λ = 7% *p* < 0.0033) (Table S3 and Figure 5A).

At 39 months, the first two canonical axes accounted for 64.2% of the variation in data, with 31.3 and 32.9% deriving from axes 1 and 2, respectively. The overall pattern was similar to that at 27 months, with a strong separation between the treatments with *A. mangium* (A and E+A) and with *Eucalyptus* (E and E+N), but with different factors and weights. The vectors that differentiated *A. mangium* (A) and mixed planting (E+A) were the microbial indicators Cmic (λ = 3%, *p* < 0.0490), Nmic (λ = 2%, *p* < 0.0478), and *q*Mic-C (λ = 1%, *p* < 0.0579), which had a marked influence (positive correlation) on soil and litter attributes such as N-OF (λ = 19%, *p* < 0.0027), C-OF (λ = 7%, *p* < 0.0201), TOC (λ = 4%, *p* < 0.0309), and Total-N (Litter) (λ = 2%, *p* < 0.0478). On the other hand, the attributes that contributed most to differentiation of E and E+N treatments were the microbial indicators *q*CO_2_ (λ = 16%, *p* < 0.0028), amidase (λ = 9%, *p* < 0.0147), dehydrogenase (λ = 9%, *p* < 0.0147), and urease (λ = 2%, *p* < 0.0489), which presented a positive correlation with litter attributes such as ${\text{NH}}_{4}^{+}$-N (λ = 9%, *p* < 0.0027) and C/N ratio (λ = 1%, *p* < 0.0501) (Table S3 and Figure 5B).

## Discussion

Our results demonstrated that soil microbial indicators associated with C and N cycles had significant changed in pure and intercropped treatments of *A. mangium* in the first rotation of plantations, even in the juvenile phase (27 and 39 months). Overall, our experiments demonstrate an improvement in soil quality (soil health), confirming our hypothesis that introduction of *A. mangium* in an intercropped *Eucalyptus* forest plantation promotes changes in microbial indicators (microbial, enzymatic, and metabolic activity) and increases in C and N concentrations in soil OM labile fractions.

### Intercropped systems exhibit increased C and N microbial contents

The soil microbial community is highly sensitive to changes in management practices and therefore represents a good indicator of soil quality (Cardoso et al., 2013). The highest concentrations of C and N microbial content in the A and E+A (Figures 2A,B) suggest a high efficiency of microbial biomass in incorporating C and N, and improved OM cycling in mixed systems with *A. mangium* from 27 months after planting. This phenomenon may be due to *A. mangium* systems depositing N-rich sources (low C/N ratio), which can accelerate the microbial attacks, enhancing labile nutrients amounts in the soil (Bini et al., 2013). Such response is relevant under sandy soil conditions, since microbial biomass (MB) represents the most labile fraction of soil OM, acting directly on the availability of nutrients important during the initial development of plants, thereby reducing the need for mineral fertilizers. Through an experiment with a similar experimental design to ours and evaluating a second rotation of pure and mixed *E. grandis* and *A. mangium* plantations, Bini et al. (2013) found that MB operated as a C drain until 14 months after planting, with a high C cycling rate at 20 months, and thus acted as a source of C in the soil. Their results suggested that the presence of *A. mangium* provides more appropriate conditions for increases in MB, a conclusion confirmed by our study. However, they did not address other key periods of forest development such as the transitional period from biochemical to biogeochemical cycling (27 and 39 months, respectively). Moreover, their study also failed to investigate the effect of changes in soil OM fractions caused by the insertion of *A. mangium*.

### Dynamics of microbial and enzymes associated with C and N cycling in soil

Enzymes act in the soil by degrading OM, promoting the mineralization of nutrients through hydrolytic and oxidative reactions, and their potential regulated among other factors by the nutritional conditions in the soil (Mendes et al., 2013). The higher potential activity of urease and amidase we identified in E and E+N treatments (Figures 3A,D) may be related to a low litter chemical quality (high C/N ratio; Table 2) and resulting increase in enzymatic production to promote material cycling. On the other hand, the low litter C/N ratio leads to a higher presence of easily hydrolysable N substrates and reduces the requirement of specific enzymes that make them available in the soil (Tabatabai, 1994), as observed in *A. mangium* treatments. It is possible that the urease and amidase enzymes play a key role in the availability of N in plantations that deposit a nutrient-poor litter, such as *E. grandis* pure forests. However, this mechanism requires further investigations into the status of N mineralization rates, since a low correlation was detected between the potential activity of these enzymes and N fractions in the soil. Additionally, the positive correlation between levels of *q*CO_2_ and dehydrogenase activity suggests stressful conditions (Figure 5), resulting in lower effectiveness of the soil microbial community at storing organic C on microbial biomass, and in higher *q*CO_2_. In this case, high dehydrogenase activity in E and E+N stands may be indicative of a more intense electron flow due to respiration under adverse conditions, in agreement with a higher *q*CO_2_ index (Casida et al., 1964; Cardoso et al., 2013). This would imply that the dynamics of nutrient cycling in pure *Eucalyptus* systems is considerably different from when *Acacia* is present.

### *A. mangium* increase C and N concentrations in the soil organic fraction

Treatments A and E+A are associated with changes in soil OM fractions even in the short run (27 and 39 months after planting). Organic matter has a key effect on physical, chemical and biological soil attributes, especially in sandy soils where it can improve soil structure, cation exchange capacity, and nutrient availability (Blanchart et al., 2005; Laclau et al., 2010). Therefore, a better understand of main microbial indicators and their correlation with C and N dynamics in soil may allow a more precise forecast of the effects of forest management on soil functions (Bini et al., 2014).

The highest contents of OF in *A. mangium* stands (A and A+E) suggested a potential longer maintenance of *Acacia* residues in the labile fraction of soil OM when compared to *Eucalyptus* treatments. Bachega et al. (2016) tested the “Home Field Advantage” hypothesis and examined the effect of litter chemistry on the decomposition of leaves and fine roots of *A. mangium* and *E. grandis*, but did not examine the effects of soil microbial community and their correlation with soil OM fractions. However, the showed that decomposition rates were slower in *A. mangium* litter than in *Eucalyptus*, even though initial N and P concentrations were higher in *A. mangium* residues. This demonstrates that litter decomposition depends partially on the C quality of the litter, primarily in terms of water-soluble compounds and lignin content. In addition, the OF has a chemical composition comparable to plant material, which may be quickly affected by fluctuations in the quality and quantity of organic inputs due to land-use change (Koutika et al., 2001), alterations in the management practice (Brandani et al., 2017), or longer periods in forest systems (Epron et al., 2015), being therefore used as a quality indicator of soil because can change rapidly the microbial community.

The significant increase in N and C contents of OF in A and E+A treatments is possibly related to the N fixation process performed by diazotrophic bacteria associated with the roots of *A. mangium* (Bouillet et al., 2008; Fonseca et al., 2017), which is corroborated by the higher N content in the litter and also higher N mineralization potential (${\text{NH}}_{4}^{+}$-N) (Voigtlaender et al., 2012). *A. mangium* is a nitrogen-fixing tree recognized for improving soil nutrient status, mostly due to its N-enriched litter (Voigtlaender et al., 2012; Epron et al., 2016; Koutika and Mareschal, 2017). Additionally, the higher N input promoted by N-fixing tree species such as *A. mangium* is directly related to the accumulation of C in the soil, mainly because they exhibit significant increases in the volume of deposited litter and fine roots produced (Binkley, 2005). Thus, the positive correlation between C and N in soil and litter, in both seasons, and in treatments A and E+A (Figures 5A,B), indicates that the higher levels of C (especially in the organic fraction of OM) are associated with a higher N concentration in these treatments.

The treatment with N addition (E+N) promoted an increase in the content of microbial N (Nmic) in both sampling times (Figure 2) but did not necessarily influence the Total-N content of the soil (Table 1). This result demonstrates that the application of soluble nutrient sources can influence soil attributes superficially, without reflecting nutrient accumulation at least in the short term. The same applies to accumulation of C in soil, where the E+N treatment did not reflect the increase of total C. Binkley (2005) points out that the increase of C in the soil by means of mixed systems is not mediated by a single and strictly chemical process, since the addition of N via fertilization may inhibit the enzymatic functions of the microbial community, especially those associated with lignin degradation (Binkley et al., 2004).

### Soil microbial indicators are influenced by climatic conditions and plant age

The study of microbial indicators and associated metabolic process under field conditions is a huge challenge, due to the dynamics of the system in the edaphoclimatic changes along a forest rotation. For example, the reduction in microbial C and N contents, *q*Mic-C index, as well as the potential activity of β-glucosidase (A treatment) and dehydrogenase enzymes at 39 months, may have been influenced by soil water stress, since a water deficit of −3 mm was observed in this period (Figure S3). The resulting increases in CO_2_-C emissions, as well as a higher *q*-CO_2_ index at 39 months, may have stressed the microbial community and reduced its efficiency at this sampling time. The reduction in the community niche (CN) index in treatments A and E+A at 39 months may have occurred for the same reason. The larger the community niche, the more efficiently a community can exploit available resources (Salles et al., 2009). Thus, in regions with prolonged water deficit throughout the year, mixed plantation systems of *E. grandis* and *A. mangium* can present low CN index and inefficiently exploit available soil resources, paralyzing or reducing the dynamics of C and N in the system.

In addition to water deficit, another relevant factor behind variation among treatments was forest age. At 39 months, plantations generally reach the maximum of litter deposit and increase nutrient cycling in soil (Laclau et al., 2010), which may justify the significant increase in microbial attributes at this stage. In addition, the high values of potential activity of amidohydrolases enzymes at 39 months may also be associated, with the greater contribution of fresh litter to the soil. This may temporarily decrease the efficiency of C use in the soil due to the scarcity of N as energy sources to the microbiota, promoting a stimulus to the activity of these enzymes, degradation of newly deposited substrates (López et al., 2012), and greater immobilization of N in the microbial biomass during this period (Figure 2B). It is known that degrading enzymes such as amidohydrolases are strongly regulated by substrate availability (Sinsabaugh, 2010).

It is worth noting that mixed treatment (E+A) showed no differences in total C and N concentrations over time (Table 1). Nonetheless, it is possible that organomineral fractions, such as N-OMinF2 and F3, as well as C-OMin-F2, have increased over time (Table 1). This means that part of the organic fraction of OM is being converted into more stable fractions of OM in the mixed plantations, slowing down microbial attacks and generating negative loads responsible for increased water retention and nutrient availability in sandy soils (Neumann et al., 2014).

## Conclusions

The use of nitrogen-fixing trees such as *A. mangium* in mixed plantation systems with *E. grandis* promoted significant changes in microbial attributes and had a strong effect on C and N dynamics in the soil, even in juvenile stages of growth (27 and 39 months). The higher concentrations of C and N in the microbial biomass demonstrate that mixed plantations promote a more efficient use of those elements by microbial communities. Moreover, microbial changes influenced soil OM fractions, especially the more labile organic fraction (OF, 2,000–75 μm) that significantly increased in the mixed plantations, thereby confirming our hypotheses. In this sense, the higher concentrations of C and N in the organic fraction of mixed plantations indicates this type of planting increases nutrient availability in soils with low levels of OM such as Latosols, commonly found in tropical regions. The areas of monoculture of *Eucalyptus*, either fertilized with N or not, present litters with high C/N ratio, resulting in higher values of metabolic quotient and dehydrogenase enzyme in the soil, which provided strong evidence for the presence of a microbial biomass with low resource efficiency. Thus, future research is needed to characterize the importance of soil microbiome in areas with pure and mixed plantations under different soil and climate conditions. This type of initiative will be necessary to reduce (partial or complete) the mineral fertilizers use in Eucalyptus plantations, so that mixed plantations may become a more generalized practice, for being more economical and sustainable.

## Author contributions

AP: contributed to the assembly of the experiment, sampling, microbial-soil-litter and statistical analyses, and writing this work; MZ: contributed sampling collection and statistical analysis; CB: contributed to the assembly of the experiments and writing this work; DM: contributed to the microbial and enzyme activity analyses; SC: contributed to the Biolog analyses; JG: contributed to the conception and review of this work; EC: contributed to the conception, orientation, writing, and review of this work.

### Conflict of interest statement

The authors declare that the research was conducted in the absence of any commercial or financial relationships that could be construed as a potential conflict of interest.
