# Supplementary material for: Acacia Changes Microbial Indicators and Increases C and N in Soil Organic Fractions in Intercropped Eucalyptus Plantations
Source: Front Microbiol. 2018 Apr 4;9:655. doi: 10.3389/fmicb.2018.00655 (PMC5893836; doi:10.3389/fmicb.2018.00655)
Supplement: Supplementary file 1 [file Image1.pdf]

Supplementary Material

Acacia changes microbial indicators and increases C and N in soil organic fractions in intercropped *Eucalyptus* plantations

Arthur P.A. Pereira[1*], Maurício R.G. Zagatto[1], Carolina B. Brandani[2], Denise de L. Mescolotti[1], Simone Raposo Cotta[1], José L.M. Gonçalves[2], Elke J.B.N. Cardoso[1*]

[1]Soil Microbiology Laboratory, "Luiz de Queiroz" College of Agriculture - University of São Paulo, Department of Soil Science, Piracicaba, Brazil

[2]"Luiz de Queiroz" College of Agriculture - University of São Paulo, Department of Forest Sciences, Piracicaba, Brazil

***Corresponding author:** Elke JBN Cardoso, Email: ejbncard@usp.br; Arthur PA Pereira, Email:

arthur.prudencio@usp.br

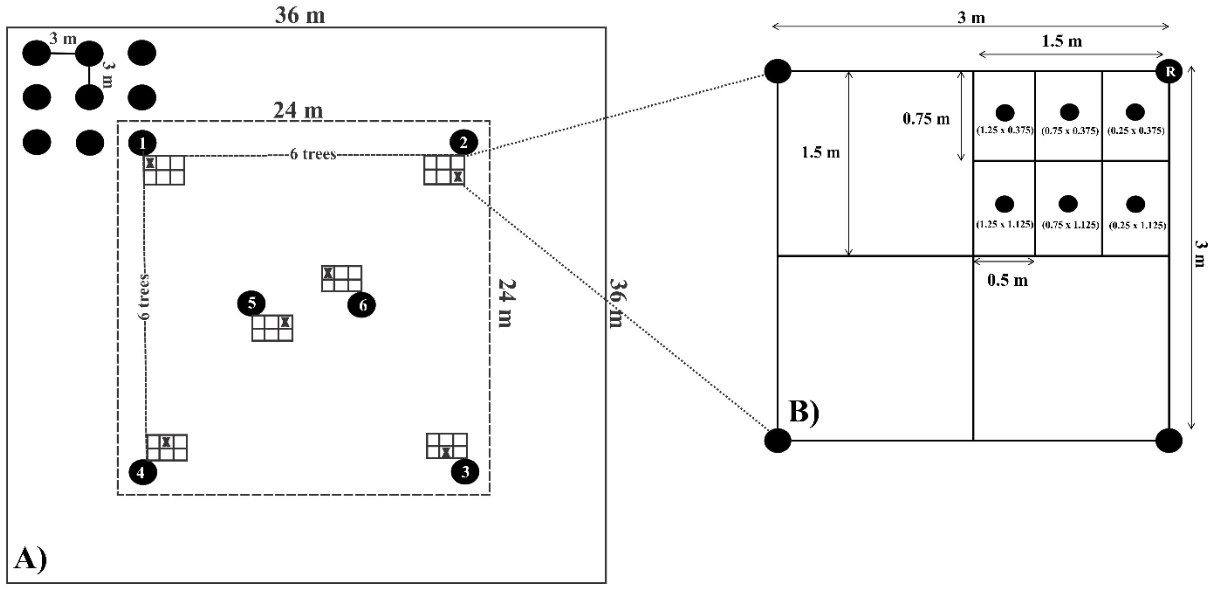

Supplementary Figure S1. Soil and litter sampling based on Voronoi grids. A) total plot area (36 x 36 m), the useful plot (24 x 24 m) and spacing between plants (3 x 3 m). Numbers 1 to 6 indicate the trees selected for sampling and "X" indicts the collection points at each tree; B) "R" represents the reference tree and the six rectangles represent the dimensions of each sampling point.
