# Supplementary material for: Acacia Changes Microbial Indicators and Increases C and N in Soil Organic Fractions in Intercropped Eucalyptus Plantations
Source: Front Microbiol. 2018 Apr 4;9:655. doi: 10.3389/fmicb.2018.00655 (PMC5893836; doi:10.3389/fmicb.2018.00655)
Supplement: Supplementary file 2 [file Image2.pdf]

Supplementary Material

Acacia changes microbial indicators and increases C and N in soil organic fractions in intercropped *Eucalyptus* plantations

Arthur P.A. Pereira[1*], Maurício R.G. Zagatto[1], Carolina B. Brandani[2], Denise de L. Mescolotti[1], Simone Raposo Cotta[1], José L.M. Gonçalves[2], Elke J.B.N. Cardoso[1*]

[1]Soil Microbiology Laboratory, "Luiz de Queiroz" College of Agriculture - University of São Paulo, Department of Soil Science, Piracicaba, Brazil

[2]"Luiz de Queiroz" College of Agriculture - University of São Paulo, Department of Forest Sciences, Piracicaba, Brazil

***Corresponding author:** Elke JBN Cardoso, Email: ejbncard@usp.br; Arthur PA Pereira, Email:

arthur.prudencio@usp.br

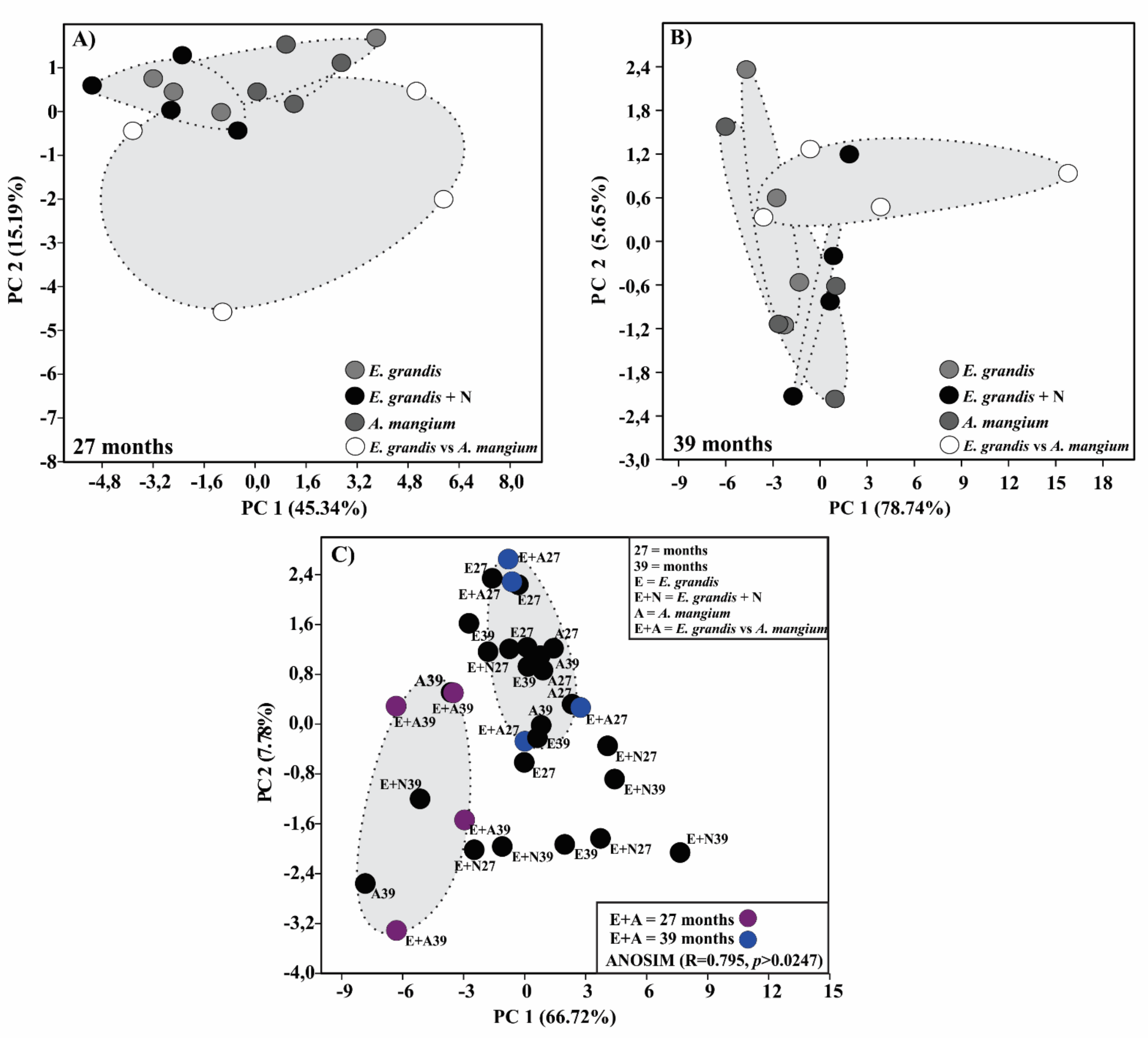

Supplementary Figure S2. Principal Coordinates Analysis (PCoA) based on the metabolic profile degradation of the soil microbial community accessed by Biolog EcoPlates in pure and mixed *E. grandis* and *A. mangium* plantations. A) 27 and B) 39 months after planting, and C) total profile.
