# Supplementary material for: Acacia Changes Microbial Indicators and Increases C and N in Soil Organic Fractions in Intercropped Eucalyptus Plantations
Source: Front Microbiol. 2018 Apr 4;9:655. doi: 10.3389/fmicb.2018.00655 (PMC5893836; doi:10.3389/fmicb.2018.00655)
Supplement: Supplementary file 3 [file Image3.pdf]

Supplementary Material

Acacia changes microbial indicators and increases C and N in soil organic fractions in intercropped *Eucalyptus* plantations

Arthur P.A. Pereira[1*], Maurício R.G. Zagatto[1], Carolina B. Brandani[2], Denise de L. Mescolotti[1], Simone Raposo Cotta[1], José L.M. Gonçalves[2], Elke J.B.N. Cardoso[1*]

[1]Soil Microbiology Laboratory, "Luiz de Queiroz" College of Agriculture - University of São Paulo, Department of Soil Science, Piracicaba, Brazil

[2]"Luiz de Queiroz" College of Agriculture - University of São Paulo, Department of Forest Sciences, Piracicaba, Brazil

***Corresponding author:** Elke JBN Cardoso, Email: ejbncard@usp.br; Arthur PA Pereira, Email:

arthur.prudencio@usp.br

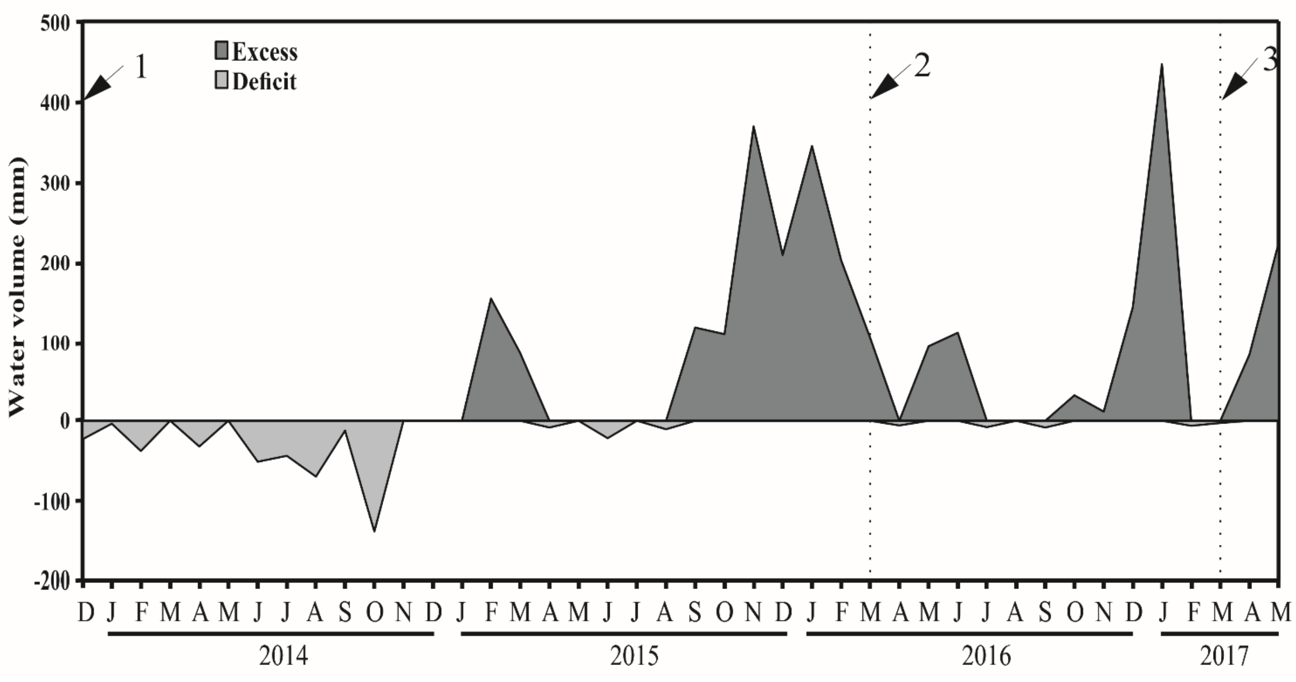

Supplementary Figure S3. Monthly sequential water balance by the method proposed by Thornthwaite and Mather (1955) during the experiment (arrow 1, December 2013) from 2014 to 2017. Arrows 2 and 3 represent the 1st (27 months) and 2nd (39 months) sampling period. Soil water storage capacity = 280 mm.

.
