# Supplementary material for: Acacia Changes Microbial Indicators and Increases C and N in Soil Organic Fractions in Intercropped Eucalyptus Plantations
Source: Front Microbiol. 2018 Apr 4;9:655. doi: 10.3389/fmicb.2018.00655 (PMC5893836; doi:10.3389/fmicb.2018.00655)
Supplement: Supplementary file 4 [file Image4.pdf]

Supplementary Material

Acacia changes microbial indicators and increases C and N in soil organic fractions in intercropped *Eucalyptus* plantations

Arthur P.A. Pereira[1*], Maurício R.G. Zagatto[1], Carolina B. Brandani[2], Denise de L. Mescolotti[1], Simone Raposo Cotta[1], José L.M. Gonçalves[2], Elke J.B.N. Cardoso[1*]

[1]Soil Microbiology Laboratory, "Luiz de Queiroz" College of Agriculture - University of São Paulo, Department of Soil Science, Piracicaba, Brazil

[2]"Luiz de Queiroz" College of Agriculture - University of São Paulo, Department of Forest Sciences, Piracicaba, Brazil

***Corresponding author:** Elke JBN Cardoso, Email: ejbncard@usp.br; Arthur PA Pereira, Email: arthur.prudencio@usp.br

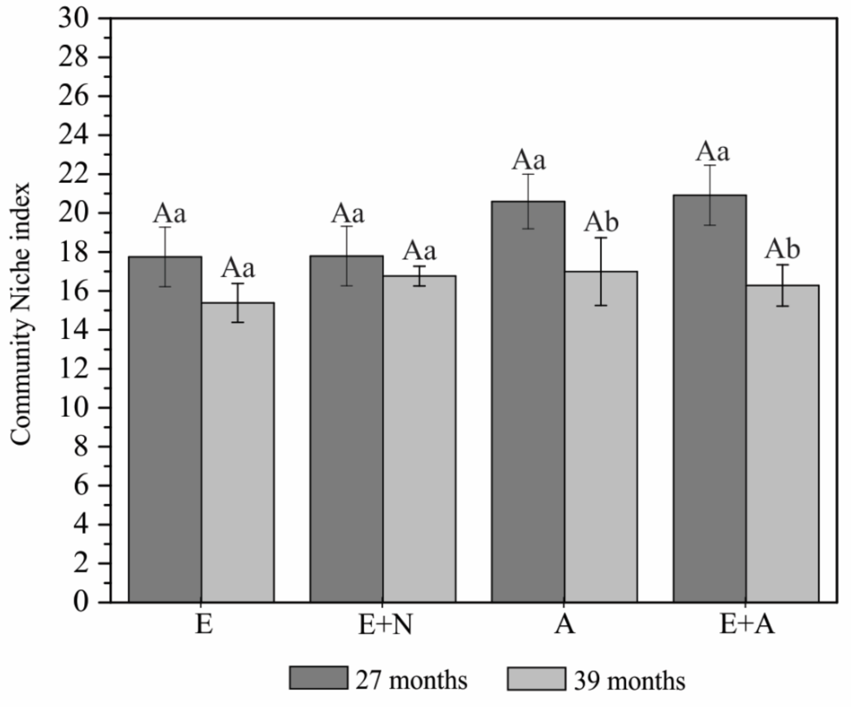

Supplementary Figure S4. Community Niche index based on the metabolic profile degradation by the soil microbial community in pure and mixed *E. grandis* and *A. mangium* plantations. (E) *E. grandis*, (E+N) *E. grandis* with N fertilization, (A) *A. mangium* and (E+A) mixed plantation between *E. grandis* and *A. mangium* at 27 and 39 months after planting. Means followed by the same letter do not differ by Tukey's test at a significance level of 5%. Upper case letters compare treatments within each period and lower-case letter compare the periods within each treatment. Error bars indicate standard deviation; *n* = 4.
