# Supplementary material for: Acacia Changes Microbial Indicators and Increases C and N in Soil Organic Fractions in Intercropped Eucalyptus Plantations
Source: Front Microbiol. 2018 Apr 4;9:655. doi: 10.3389/fmicb.2018.00655 (PMC5893836; doi:10.3389/fmicb.2018.00655)
Supplement: Supplementary file 5 [file Table1.DOCX]

Table S1. Soil correctives used in the *E. grandis* pure treatments (E and E+N), (A) *A. mangium* and (E+A) mixed plantation between *E. grandis* and *A. mangium*.

|  | 2013 | | | | | | 2014 | |
| --- | --- | --- | --- | --- | --- | --- | --- | --- |
|  | N | K_2_O | P_2_O_5_ | Micro. | Limestone | B | N | K_2_O |
|  | -----------------------------------kg ha^-1^----------------------------------- | | | | | | | |
| E | - | 30 | 100 | 30 | 2000 | 4.5 | - | 120 |
| E+N | 10 | 30 | 100 | 30 | 2000 | 4.5 | 90 | 120 |
| A | - | 30 | 100 | 30 | 2000 | 4.5 | - | 120 |
| E+A | - | 30 | 100 | 30 | 2000 | 4.5 | - | 120 |

Sources:

N: Ammonium sulfate.

K_2_O: Potassium Chloride.

P_2_O_5_: Triple Superphosphate.

Micronutrients: FTE BR12 - granulate.

Limestone: Dolomitic to supply Ca^2+^ and Mg^2+^.

B: Boron/Borogran®.
