# Supplementary material for: Acacia Changes Microbial Indicators and Increases C and N in Soil Organic Fractions in Intercropped Eucalyptus Plantations
Source: Front Microbiol. 2018 Apr 4;9:655. doi: 10.3389/fmicb.2018.00655 (PMC5893836; doi:10.3389/fmicb.2018.00655)
Supplement: Supplementary file 6 [file Table2.DOCX]

|  |  | **E** | | **E+N** | | **A** | | **E+A** | |
| --- | --- | --- | --- | --- | --- | --- | --- | --- | --- |
|  | **C sources** | **27** | **39** | **27** | **39** | **27** | **39** | **27** | **39** |
|  |  | Average Well Colour Development (AWCD) | | | | | | | |
| **I** | α-D-lactose | 0.36 | 0.33 | 0.37 | 0.40 | 0.39 A | 0.32 B | 0.42 A | 0.34 B |
|  | D-cellobiose | 0.40 | 0.41 | 0.37 | 0.50 | 0.40 | 0.45 | 0.42 | 0.44 |
|  | D-mannitol | 0.79 | 0.89 | 0.79 | 0.85 | 0.96 A | 0.71 B | 0.96 | 0.78 |
|  | D-xylose | 0.49 | 0.53 | 0.41 | 0.43 | 0.43 A | 0.35 B | 0.44 A | 0.37 B |
|  | i-erythritol | 0.42 | 0.48 | 0.43 | 0.40 | 0.46 | 0.35 | 0.45 | 0.34 |
|  | *N*-acetyl-D-glucosamine | 0.65 | 1.18 | 0.62 | 0.62 | 0.041 B | 0.64 A | 0.70 | 0.71 |
|  | β-methyl-D-glucoside | 0.43 | 0.48 | 0.38 | 0.53 | 0.40 | 0.76 | 0.42 | 0.57 |
| **II** | α-cyclodextrin | 0.42 | 0.48 | 0.37 | 0.39 | 0.40 A | 0.32 B | 0.45 | 0.34 |
|  | Glycogen | 0.42 | 0.42 | 0.38 | 0.41 | 0.41 A | 0.33 B | 0.43 | 0.36 |
|  | Tween 40 | 0.74 | 0.98 | 0.82 | 0.91 | 0.86 A | 0.69 B | 0.83 | 0.80 |
|  | Tween 80 | 0.84 | 1.01 | 0.69 | 0.77 | 0.73 | 0.63 | 0.84 | 0.63 |
| **III** | 2-hydroxy benzoic | 0.42 | 0.48 | 0.36 | 0.43 | 0.39 A | 0.32 B | 0.41 | 0.34 |
|  | 4-hydroxy benzoic | 0.56 | 0.65 | 0.73 | 0.61 | 0.60 | 0.47 | 0.62 | 0.57 |
|  | α-ketobutyric | 0.39 | 0.42 | 0.38 | 0.39 | 0.40 A | 0.32 B | 0.42 | 0.34 |
|  | D-galactonic γ-lactone | 0.88 | 0.97 | 0.74 | 0.76 | 0.77 A | 0.60 B | 0.76 | 0.67 |
|  | D-galacturonic | 0.43 | 0.50 | 0.75 | 0.57 | 0.71 | 0.48 | 0.69 A | 0.35 B |
|  | D-glucosamic | 0.66 | 0.66 | 0.62 | 0.60 | 0.68 A | 0.47 B | 0.69 | 0.54 |
|  | D-malic | 0.39 | 0.37 | 0.39 | 0.42 | 0.39 | 0.37 | 0.43 | 0.40 |
|  | γ-hydroxy butyric | 0.46 | 0.51 | 0.49 | 0.45 | 0.52 | 0.43 | 0.52 | 0.36 |
|  | Itaconic Acid | 0.58 | 048 | 0.50 | 0.50 | 0.60 | 0.43 | 0.61 | 0.44 |
| **IV** | Phenylethyl-amine | 0.41 | 0.48 | 0.44 | 0.46 | 0.42 | 0.37 | 0.47 | 0.45 |
|  | Putrescine | 0.69 | 0.67 | 0.69 | 0.72 | 0.69 A | 0.56 B | 0.42 | 0.47 |
| **V** | Glycyl-L-glutamic | 0.42 | 0.46 | 0.39 | 0.41 | 0.41 A | 0.33 B | 0.44 A | 0.35 B |
|  | L-arginine | 0.83 | 0.93 | 0.86 | 0.78 | 0.82 | 0.64 | 0.82 | 0.78 |
|  | L-asparagine | 0.80 | 1.03 | 0.97 | 0.95 | 0.98 A | 0.72 B | 0.96 | 0.89 |
|  | L-phenylalanine | 0.51 | 0.56 | 0.43 | 0.45 | 0.46 | 0.40 | 0.49 | 0.45 |
|  | L-serine | 0.64 | 0.95 | 0.87 | 0.83 | 0.80 | 0.78 | 0.84 | 0.79 |
|  | L-threonine | 0.49 | 0.49 | 0.37 | 0.40 | 0.40 A | 0.33 B | 0.43 A | 0.35 B |
| **VI** | D,L-α-glycerol phosphate | 0.51 | 0.48 | 0.40 | 0.45 | 0.47 | 0.39 | 0.46 | 0.36 |
|  | Glucose-l-phosphate | 0.39 | 0.47 | 0.42 | 0.41 | 0.39 A | 0.31 B | 0.42 | 0.39 |
|  | Pyruvic acid methyl ester | 0.66 | 0.81 | 0.58 | 0.56 | 0.61 | 0.59 | 0.65 | 0.52 |

Table S2. Metabolic profile assessed by the Biolog EcoPlates in pure and mixed *E. grandis* and *A. mangium* plantations. (E) *E. grandis*, (E+N) *E. grandis* with N fertilization, (A) *A. mangium* and (E+A) mixed plantation of *E. grandis* and *A. mangium* at 27 and 39 months after planting.

.

I: Carbohydrates; II: Polymers; III: Carboxylic acids; IV: Amines; V: Amino acids; VI: Miscellaneous.
