# Supplementary material for: Acacia Changes Microbial Indicators and Increases C and N in Soil Organic Fractions in Intercropped Eucalyptus Plantations
Source: Front Microbiol. 2018 Apr 4;9:655. doi: 10.3389/fmicb.2018.00655 (PMC5893836; doi:10.3389/fmicb.2018.00655)
Supplement: Supplementary file 7 [file Table3.DOCX]

Table S3. Percentage of variation in soil microbiological patterns between treatments correlated with the soil and litter parameters based on Monte Carlo permutation test.

|  | Conditional effects | | | |
| --- | --- | --- | --- | --- |
| Attributes | Lambda  (λ) | Contribution (%) | F-test | *p* value |
| 27 months |  |  |  |  |
| Total-N (Litter) | 0.17 | 17 | 4.27 | 0.0019 |
| C-OF | 0.14 | 14 | 3.27 | 0.0017 |
| NH_4_^+^-N (Litter) | 0.10 | 10 | 5.38 | 0.0011 |
| N-OF | 0.10 | 10 | 4.89 | 0.0018 |
| N-OMF1 | 0.09 | 9 | 4.23 | 0.0010 |
| Amidase | 0.09 | 9 | 3.54 | 0.0019 |
| Dehydrogenase | 0.7 | 7 | 5.06 | 0.0014 |
| Cmic | 0.05 | 5 | 3.30 | 0.0039 |
| C/N | 0.03 | 3 | 3.29 | 0.0033 |
| CO_2_-C | 0.02 | 2 | 3.30 | 0.0039 |
| TOC | 0.02 | 2 | 3.75 | 0.0280 |
| *q*CO_2_ | 0.02 | 2 | 3.17 | 0.0441 |
| *q*Mic-C | 0.01 | 1 | 3.14 | 0.0410 |
| Urease | 0.01 | 1 | 3.01 | 0.0501 |
| L-glutaminase | - | - | 2.87 | 0.0578 |
| L-asparaginase | - | - | 2.01 | 0.0935 |
| *q*Mic-N | - | - | 1.17 | 0.1478 |
| Nmic | - | - | 1.09 | 0.2478 |
| 39 months |  |  |  |  |
| N-OMF1 | 0.21 | 21 | 3.24 | 0.0017 |
| N-OF | 0.19 | 19 | 3.06 | 0.0027 |
| *q*CO_2_ | 0.16 | 16 | 3.14 | 0.0028 |
| NH_4_^+^-N (Litter) | 0.09 | 9 | 3.93 | 0.0027 |
| Amidase | 0.09 | 9 | 3.86 | 0.0147 |
| Dehydrogenase | 0.09 | 9 | 3.13 | 0.0147 |
| C-OF | 0.07 | 7 | 3.47 | 0.0201 |
| TOC | 0.04 | 4 | 3.20 | 0.0309 |
| Cmic | 0.03 | 3 | 3.16 | 0.0490 |
| Total-N (Litter) | 0.02 | 2 | 3.91 | 0.0478 |
| L-glutaminase | 0.02 | 2 | 3.25 | 0.0421 |
| Nmic | 0.02 | 2 | 3.08 | 0.0478 |
| Urease | 0.02 | 2 | 3.19 | 0.0489 |
| L-asparaginase | 0.01 | 1 | 3.73 | 0.0407 |
| C/N | 0.01 | 1 | 3.04 | 0.0501 |
| *q*Mic-C | 0.01 | 1 | 2.98 | 0.0579 |
| *q*Mic-N | - | - | 2.24 | 0.0689 |
| CO_2_-C | - | - | 1.37 | 0.9789 |
